# High Ultraviolet Absorption in Colloidal Gallium Nanoparticles Prepared from Thermal Evaporation

**DOI:** 10.3390/nano7070172

**Published:** 2017-07-06

**Authors:** Flavio Nucciarelli, Iria Bravo, Sergio Catalan-Gomez, Luis Vázquez, Encarnación Lorenzo, Jose Luis Pau

**Affiliations:** 1 Physics Department, Lancaster University, Lancaster LA1 4YB, UK; 2 Grupo de Electrónica y Semiconductores, Departamento de Física Aplicada, Universida Autónoma de Madrid, 28049 Madrid, Spain; sergio.catalan@uam.es (S.C.-G.); joseluis.pau@uam.es (J.L.P.); 3 Departamento de Química Analítica y Análisis Instrumental, Universidad Autónoma de Madrid, Cantoblanco, 28049 Madrid, Spain; iria.bravo@uam.es (I.B.); encarnacion.lorenzo@uam.es (E.L.); 4 Instituto Madrileño de Estudios Avanzados en Nanociencia (IMDEA-Nanociencia), Faraday, 9, Campus UAM, Cantoblanco, 28049 Madrid, Spain; 5 Materials Science Factory, Instituto de Ciencia de Materiales de Madrid, CSIC, 28049 Madrid, Spain; lvb@icmm.csic.es; 6 Institute for Advanced Research in Chemical Sciences (IAdChem), Universidad Autónoma de Madrid, 28049 Madrid, Spain

**Keywords:** gallium, nanoparticle, colloid, AZO, tetrahydrofuran, thermal evaporation, DDA simulation

## Abstract

New methods for the production of colloidal Ga nanoparticles (GaNPs) are introduced based on the evaporation of gallium on expendable aluminum zinc oxide (AZO) layer. The nanoparticles can be prepared in aqueous or organic solvents such as tetrahydrofuran in order to be used in different sensing applications. The particles had a quasi mono-modal distribution with diameters ranging from 10 nm to 80 nm, and their aggregation status depended on the solvent nature. Compared to common chemical synthesis, our method assures higher yield with the possibility of tailoring particles size by adjusting the deposition time. The GaNPs have been studied by spectrophotometry to obtain the absorption spectra. The colloidal solutions exhibit strong plasmonic absorption in the ultra violet (UV) region around 280 nm, whose width and intensity mainly depend on the nanoparticles dimensions and their aggregation state. With regard to the colloidal GaNPs flocculate behavior, the water solvent case has been investigated for different pH values, showing UV-visible absorption because of the formation of NPs clusters. Using discrete dipole approximation (DDA) method simulations, a close connection between the UV absorption and NPs with a diameter smaller than ~40 nm was observed.

## 1. Introduction

Insulating, metal and semiconducting nanoparticles (NPs) have played a crucial role in the development of nanotechnology during the last two decades. The driving force in the field comes from the large surface-to-volume ratio and the manifestation of quantum effects that make the physical and chemical properties of these particles different from those found in bulk materials. Due to their applicability in different areas such as drug delivery [1], disinfection [2], photovoltaics [3], (bio)sensing [4,5], and catalysis [6,7], many studies have addressed the need for reproducible and reliable methods for the preparation of NPs. One of the main goals has been the production of colloidal suspensions in a liquid medium to ease the manipulation, storage, chemical modification and administration of the NPs in many practical cases.

Metal nanoparticles can be prepared as colloidal suspensions following bottom-up or top-down approaches. After Turkevich’s work in the early fifties on the synthesis of gold NPs in aqueous solution at boiling temperature, one of the most popular bottom-up methods for producing metal NPs became the reduction of metal salts solved in a liquid phase [8,9]. Other liquid-phase methods commonly used for the fabrication of metal NPs have been magnetic-microwave heating [10], template synthesis [11] and electrodeposition [12]. They typically require the use of toxic reagents, which remain adsorbed on the surface after the NP synthesis and have potential adverse effects on human health [13]. On the other hand, gas phase methods are based on the nucleation and condensation of the gas molecules after supersaturation [14]. Examples of these methods are laser ablation, spray pyrolysis and furnace evaporation. Generally, size-selective precipitation is needed in liquid and gas phase methods to produce narrow size distributions [15].

Metal NPs are highly soluble in nonpolar organic solvents where, due to the low dielectric constant of the medium, the solvent molecules are not able to screen the surface charges around the particle, keeping a repulsive force between the NPs [16]. However, in polar solvents, the electrostatic surface interactions between the particles tend to produce their condensation and precipitation, giving rise to disordered aggregates that are not easily resoluble [17]. Those interactions are dominated by Van der Waals forces that increase inversely with the sixth power of distance between them [18]. The light-cluster interaction will depend on the number of agglomerated NPs, their special arrangement and the surface-environment interplay [19]. For that reason, the aqueous colloids must be stabilized to reduce surface interactions between NPs through the formation of self-assembled monolayers, polymeric coating or the use of ionic liquids [20,21]. The stabilization of the NPs is therefore considered a key task in the preparation of NPs, and the steps followed to achieve this are intimately involved in the fabrication process.

Gallium (Ga) is a low melting point metal whose interesting properties have attracted much attention in recent years. Colloidal NPs of this material with sizes lower than 50 nm have been characterized, exhibiting strong plasmonic absorption bands in the ultraviolet region [22,23], which makes them very attractive for surface-enhanced Raman scattering and fluorescence spectroscopy under UV excitation. They are in liquid state at room temperature surrounded by a thin gallium oxide layer that keeps them stable for months at ambient conditions [23]. The NPs have also shown a large charge storage capacity, fostering their investigation as one of the components in lithium ion batteries. Thanks to their fast reaction in the acidic environment found around tumors, they have also performed optimally for drug delivery purposes in cancer treatment [24]. Indeed, in vivo experiments in mice have demonstrated the reduction of tumors through the release of doxorubicin in the affected area without clear signs of toxicity. For this application, the size of the NPs must be very specific to allow surpassing of the cell barrier and delivery of the drug inside the cell to provoke the apoptosis of the tumoral cells.

Monodisperse colloidal Ga NPs have been synthesized using Ga alkylamides precursors in liquid-phase chemistry [23]. Another method based on the co-deposition of Ga atoms and solvent molecules at 77 K has been reported, yielding colloidal NPs in different organic solvents like tetrahydrofuran (THF), 2-propanol and acetone [22]. Furthermore, taking advantage of the liquid state of the Ga-In eutectic alloy, ultrasound sonication of bulk pieces has also enabled the production of large concentrations of NPs in a controlled manner [24]. In this work, we propose a bottom-up method for the production of GaNP colloids from the physical vapor deposition (PVD) of Ga under vacuum conditions on a solid substrate that contains a metal oxide expendable layer. The method provides a coating layer of self-assembled NPs without photolithographic steps.

The fabrication process enables the synthesis of colloids with a quite uniform NP size in polar protic and aprotic solvents after etching the oxide layer in an acidic solution. The aggregation of the NPs in THF and aqueous solutions is investigated through ultraviolet/visible spectrophotometry. The Ga NPs absorbance spectrum is usually found in the UV range [2] and it has its origin in the high plasmon frequency of the material. When a photon with the resonance energy impinges on the particle, a localized surface wave is built and the resultant energy can be absorbed or reemitted as scattered light. Additionally, the wave gives rise to a near-field enhancement around the structure. The nanoparticle size distribution can be tuned by changing the evaporation time of the gallium target. Furthermore, the use of physical vapor deposition methodologies reduces the cost, improves the reproducibility of the NP preparation, and enables pre-functionalization and size tuning separately from the selected solvent. The method can be easily scaled to large coverage areas to increase the fabrication yield. Moreover, in comparison to other methods, it produces a minimum amount of contaminant wastes.

## 2. Results and Discussion

### 2.1. Colloidal Synthesis Optimization

**Figure 1:**
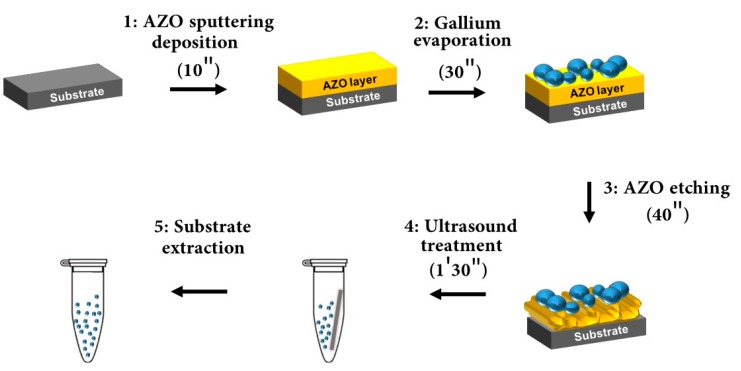
Colloidal Ga NPs synthesis. Step 1: about 300 nm of AZO expendable layer deposition on silicon substrate; Step 2: Ga NPs evaporation on the AZO layer; Step 3: the expendable layer is etched until NPs almost detached from the surface; Step 4: by means of ultrasound treatment, the NPs are transfer into the solution; Step 5: extraction of the substrate from the as-synthetized colloidal solution.

A colloidal solution of gallium nanoparticles was prepared following the procedure as shown in Figure 1. The step-by-step process will be analyzed in the methods and materials section, while the technological problems and the colloid opto-chemical features are presented here.

**Figure 2:**
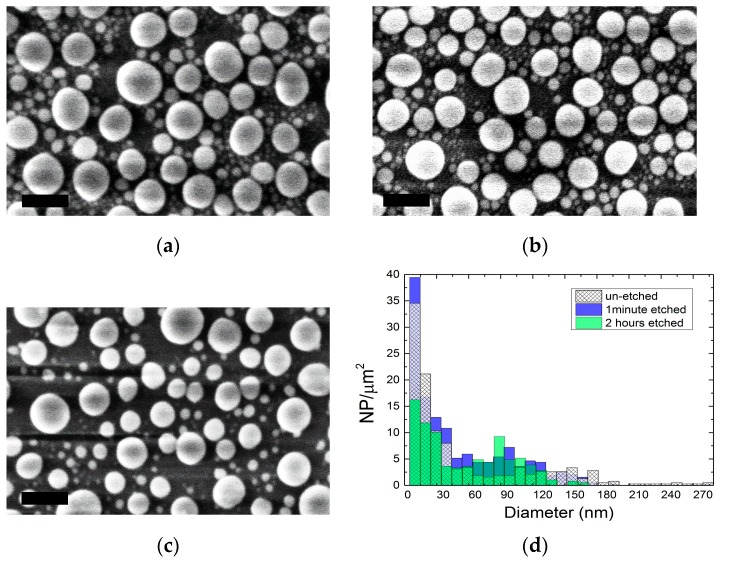
(**a**) Ga NPs evaporated on a glass substrate and immersed in an acidic bath for (**b**) 1 min; (**c**) 2 h. After 2 h, many of the smallest NPs have disappeared and the biggest ones have reduced their dimensions. (**d**) Histograms obtained from the SEM images. Scale bars are all 200 nm in length.

The endurance of the Ga nanoparticles in acidic environments was studied after their evaporation on a bare glass substrate. The substrates were immersed in a phosphoric acid/acetic acid/deionized water (1:1:75) bath for various times, ranging from 1 min to 8 h. The NP size distribution of each SEM image was determined and compared, in order to study the etching rate of the NPs. After a 1 min etch (Figure 2b) the NPs size did not change significantly, revealing a similar distribution as the un-etched sample (Figure 2a). This is a remarkable result, considering that the AZO layer has a total etching time of less than 1 min. Clearly, after 2 h (Figure 2c) small NPs started to disappear, as the biggest ones reduced their dimension, showing an increase of the number of NPs with an average size of 90 nm. After 8 h (not shown), all the NPs dissolved completely, leaving no residual of Ga on the glass surface.

In acidic medium, the NPs’ oxide shell did not endure more than a few minutes [25,26], provoking the exposure of the gallium to the chemical solution. As time passed, the metal kept oxidizing slowly, and the new oxide layers were quickly dissolved until all the inner metal gallium disappeared. The comparison between histograms of 1 min and 2 h etched samples (Figure 2d) shows a decrease in the smaller NP population. For this reason, the etching process in our synthesis has been kept under 1 min, to avoid changes in the NPs’ morphology.

**Figure 3:**
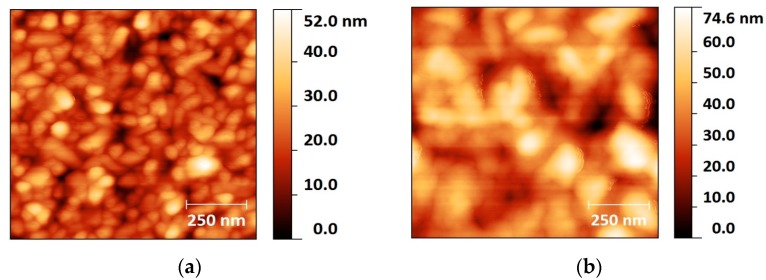
AFM images of the surface of AZO layer deposited at (**a**) RT and (**b**) 300 °C.

The AZO expendable layer in the preparation scheme (Figure 1) was deposited at room temperature (RT) and 300 °C in order to compare possible structural differences of the two surfaces and find the best conditions for the Ga NP deposition. As atomic force microscopy (AFM) analysis shows (Figure 3), the room temperature (RT) sample exhibited a mean surface roughness of 6 nm, while, for high-temperature growth, this value doubled to 13.6 nm. The images clearly show a smaller grain size for the RT layer, compared to the 300 °C sample.

**Figure 4:**
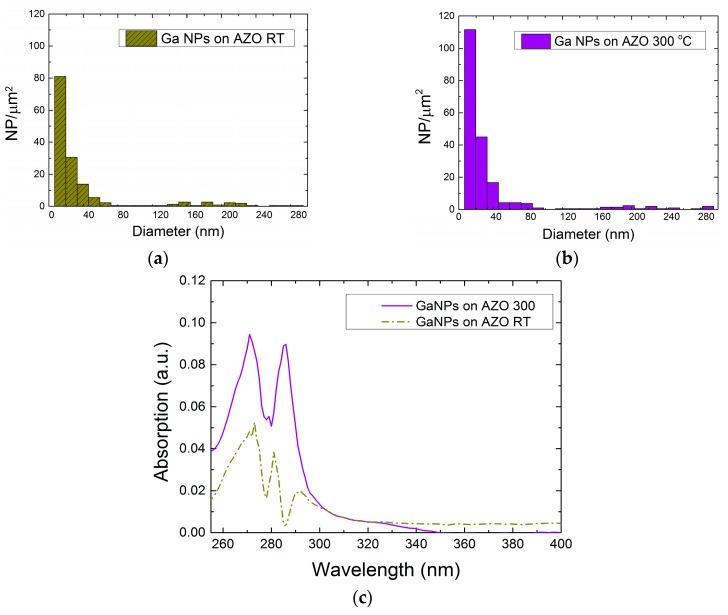
As-evaporated distributions of Ga NPs on (**a**) an AZO layer deposited at RT and (**b**) AZO layer deposited at 300 °C. (**c**) Absorption spectra in THF solvent.

In order to identify the most suitable candidate for our process, Ga was evaporated on both AZO surfaces. Figure 4a,b show the as-evaporated distributions, where the first column is centered at a diameter of 12 nm with a width of ±6 nm, and includes the largest number of NPs in both cases. Comparing the RT and 300 °C histograms, the NPs’ dimensions are not affected by the deposition temperature of the AZO, but their concentration is. In particular, the higher the AZO grown temperature, the higher the number of particles obtained per square micron. This increase in the number of particles might be related to the faceting of the AZO surface upon growth at higher temperature, and the larger surface to volume ratio in those samples.

In addition, the entire colloidal synthesis for both types of expendable layers was performed in order to study possible changes in the optical characteristics. For that aim, the absorption spectroscopy analysis between 200 nm and 1000 nm was carried out in THF solvent. A larger absorption in the UV spectra was observed for both samples (Figure 4c) and similar experiments have been conducted in sets of different AZO thickness. As expected, higher reproducibility and larger absorption was obtained in the solution prepared from the layers grown at 300 °C.

The expendable layer etching rate was also determined for AZO layers deposited at 300 °C. Layers of 300 nm were grown on silicon for that purpose. By measuring the etching depth as a function of time, the etching rate was found to be 18 nm/s. Similar samples with evaporated Ga NPs were also investigated, and the etching rate in this case was estimated to be 6 nm/s, three times slower than the uncovered case.

**Figure 5:**
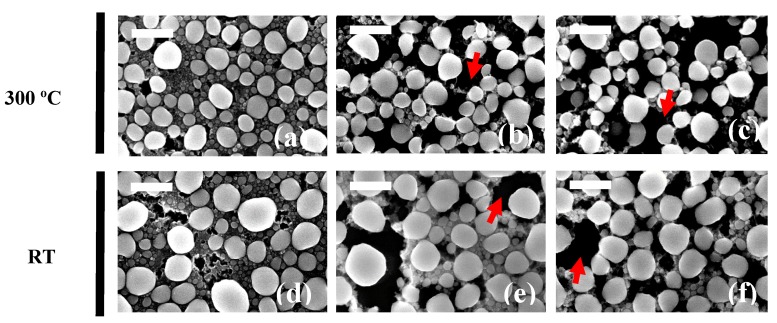
Ga NP/AZO/Si sample immersed in water/phosphoric acid/acetic acid bath for (**a**,**d**) 5 s (**b**,**e**) 15 s and (**c**,**f**) 20 s. Scale bar is 500 nm in length in all the pictures.

Further investigations of the evaporated Ga NPs on AZO layers grown at RT and 300 °C were carried out. The samples were etched in a phosphoric acid/acetic acid/deionized water (1:1:75) solution for 5 s, 15 s and 20 s (Figure 5). Scanning electron microscopy was performed to study the etching evolution of the expendable layer in order to obtain information on the minimum time to assure the NPs detachment from the hard support. As the AZO layer gradually disappears (dark zone indicated by arrows) the particles have higher probability to be dispersed in the solution by means of ultrasound treatment. After 5 s etching (Figure 5a,d), the layer was not dissolved and no significant changes were produced. Between 15 and 20 s, the NPs were less bonded to the expendable layer, and significant void areas are found between the largest NPs. Thus, the etching time for colloidal synthesis was chosen to be 40 s, in order to assure the highest NP detachment rate while preserving the NP size obtained after the evaporation.

### 2.2. Optical Characteristics of Ga NPs

In order to study the interactions between the nanoparticles and the surrounding environment, two different solvents were used to prepare the Ga NPs colloids: deionized water (DIW) and tetrahydrofuran (THF). By means of aqueous solution, we studied how colloidal Ga NPs behave at different pH levels, while tetrahydrofuran was used to test the sample in a polar aprotic environment. Table 1 shows their chemical and optical features, which have also been used to study and simulate the developed samples. The protic and aprotic nature of the solvents influences the Ga NPs agglomeration status, as will be discussed later. The refractive index values were used to simulate the stand-alone and cluster nanoparticle status in both solvents with the use of the discrete dipole approximation (DDA) method. Finally, considering that both solvents heavily absorb light in the UV range, only the data obtained above their cut-off wavelength were analyzed.

The stability of NPs in a solution depends on the interface between the surrounding media and the nanoparticle. In the case of colloids, the force balance between close particles may be expressed in consideration of different contributions such as gravitational, inertial, electrical and chemical reactivity [28]. Since the first two may be neglected for nanoparticles smaller than 0.5 µm [28,29], according to the Derjaguin, Landau, Vervey, and Overbeek (DLVO) theory [30,31] the electrostatic (repulsive) and Van der Waals (attractive) forces have a primary role in the inter-particle interaction. Recently, an extended DLVO theory (XDLVO) [32] demonstrated that the colloid’s stability also takes into account other parameters, such as the ion concentration in the solution and the NP’s hydrophobicity, which are relevant to our case of study.

#### 2.2.1. Ga NPs in THF Solvent

**Figure 6:**
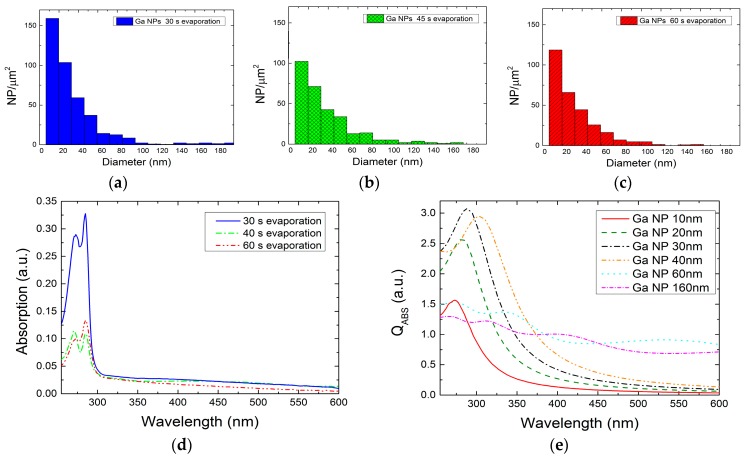
Ga NP distributions for (**a**) 30 s; (**b**) 45 s and (**c**) 60 s evaporation time; (**d**) The optical absorption was measured after colloidal preparation in THF; (**e**) Numerical simulation of the absorption efficiency (QABS) in structures with different diameters was carried out in order to find a relationship between the measured spectra and the NPs distributions.

The Ga NPs colloids in THF solution were prepared and optically characterized in the UV/visible range. Different NPs’ size distributions were found using 30 s, 45 s and 60 s evaporation time. Figure 6 shows the related histograms of the three samples obtained from scanning electron microscopy (SEM) images. In the case of 30 s evaporation, a quasi-unimodal distribution was obtained, where the density of NPs with diameters lower than 40 nm density was higher than in the other two samples. Furthermore, SEM investigation of the tilt drop-cast sample shows that our colloidal Ga NPs have a hemispherical structure similar to that obtained by other authors [33,34]. During the Ga thermal evaporation, the NP’s shape strongly depends of the nucleation process, which is related to the surface wettability of the substrate material. When the NPs’ outer surface meets oxygen, an oxide shell covers the metal core and preserves the obtained geometry. The NPs also keep this geometry after being transferred to the solvent.

The experimental absorption spectra of the three samples are shown in Figure 6d. As already discussed, THF has a cut-off absorbance wavelength of 212 nm, and the measurements were considered reliable only for data above that value. The curves have a similar shape, and exhibit a main absorption band between 260–300 nm with peaks centered at 273 nm and 285 nm. According to the Beer-Lambert’s law, the measured optical signal is strictly related with the concentration of the smallest Ga NPs in the solution, which scales well for the exhibited distributions. Bigger NPs may contribute to the broad band between 300–600 nm.

In order to further understand the relationship between the experimental absorption and the Ga NP distribution, the light-matter interaction was theoretically studied through discrete-dipole approximation implemented by DDSCAT software (Department of Astrophysical Sciences, Princeton University, U.S.) [35]. The simulations consisted of hemispherical NPs of liquid gallium, with a 2 nm native oxide shell in THF solvent. The liquid gallium and gallium oxide permittivities were extracted from Knight et al. [34] and Rebiena et al. [36], respectively, whereas solvent refractive index refers to the value in Table 1. In order to reproduce the experimental conditions, the diameter of the NPs varies between 10 and 160 nm. Their optical behavior was studied for wavelengths ranging from 255 to 800 nm. In addition, a circular polarized light beam was set to emulate the un-polarized beam from the spectrophotometer source. Interaction cut-off and error tolerance parameters were set as high as possible, with values of 3 $\times 10^{3}$ and $10^{-4}$, respectively. The simulation results were expressed in terms of absorption efficiency (Q_ABS_) and scattering efficiency (Q_SCAT_), both of which depend on the target cross-sectional area. Note that the absorption coefficient is directly proportional to the particle diameter, while the scattering is proportional to the fourth power of it. Figure 6e shows Q_ABS_ for different NP diameters, where 30 nm has the highest value in the UV region. Thus, the experimental absorption band is likely related to absorption NPs of smaller diameters (10–40 nm). The biggest NPs contribute less to the absorption at low wavelengths, but they can be responsible for the absorption at higher wavelengths, according to the simulations.

It is believed that the stability of the Ga NPs is high, and does not show agglomeration effects. The outer gallium oxide shell may confer hydrophobic behavior, preventing agglomerative clustering. Similar results have been found by Vasiliev [37] during the investigation of polymer-coated Ag nanoparticles, where different agglomeration statuses for Ag—functionalized and unfunctionalized colloidal—were verified both in water and THF.

#### 2.2.2. Ga NPs in Aqueous Solution

The behavior of the Ga NP colloids was also studied in the case of aqueous solution under different pH conditions. The acid/base level of the sample was achieved by adding NaOH $10^{-1}$ M and HCl $10^{-1}$ M solutions to reach the target acidity. Generally, the NPs’ flocculation behavior is related to their surface charge, estimated from the zeta potential (ζ) measurement. The latter calculates the electrokinetic potential of the colloid and the further the value is from zero, the less the NPs aggregate. In the case of Ga oxide NPs, the zeta potential zero value—the so-called point of zero charge (PZC)—is set between pH 7.5–8.5 [38], and because of the Ga NPs oxide shell, it was assumed that our colloidal nanoparticles would have the same PZC value.

**Figure 7:**
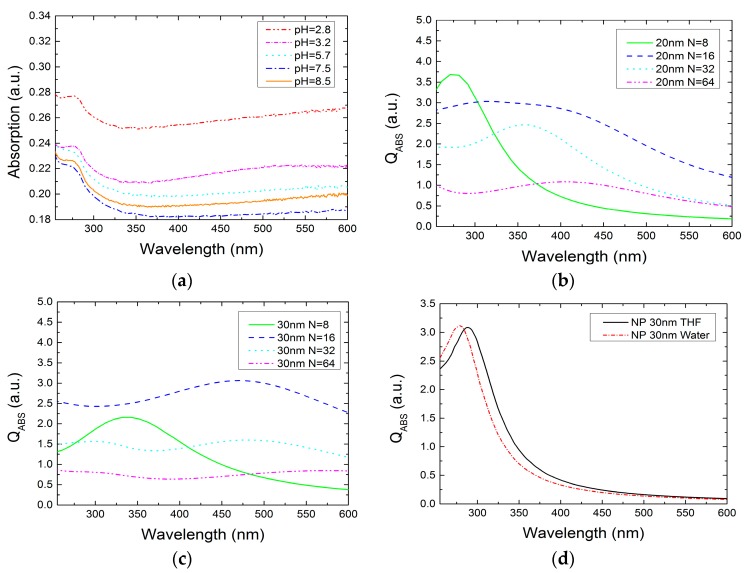
(**a**) Absorption measurements of aqueous colloidal solutions for different pH levels ranging from 2.8 to 8.5. Results of the simulations for NP clusters made of N number of agglomerated spheres with (**b**) 20 nm and (**c**) 30 nm diameter. (**d**) Calculated QABS of NPs in THF and aqueous solution.

Figure 7a shows the comparison of colloidal absorption spectra for different pHs. The experiment was carried out on 3 mL of colloidal solution ranging in pH from 2.8 to 8.5, as described in the methods section. As the pH shifts from acidic to basic, the absorption decreases when the pH approaches 7.5 (near the PZC of the Ga oxide particle), and it starts rising again at a value of 8.5. The signal tends to decrease, getting close to the PZC pH value because of cluster formation, which reduces the colloidal absorption in the observed range. All the curves show a characteristic absorption band between 250 nm and 300 nm, which is related to the colloidal Ga NP size distribution as in the THF case.

Further simulation on NP agglomeration and comparison between the aqueous and the THF case absorption peak were investigated. To evaluate the Ga NPs cluster absorption trend in aqueous solution, different structures made from 8 to 64 sphere nanoparticles were simulated by the DDA method (Figure 7b,c) [39]. Usually, the light interaction with an NP cluster generates a higher scattering contribution than absorption according to the Mie theory and both contributions are red-shifted compared to the single particle characteristic. When more than 16 nanoparticles of 20 nm diameter agglomerate, the absorption decreases drastically, as shown in Figure 7b. A similar result was found for the 30 nm nanoparticle cluster, where the maximum agglomeration level before the absorption falls was 16 NPs, as well. For this reason, the decreasing trend of the absorption for the pH change may be explained by the agglomeration theory. The closer the pH is to the PZC, the higher the agglomeration occurring between NPs, which leads to a lower absorption signal. In addition, the NP plasmon resonance condition for the aqueous and THF solutions was compared. As expected, the nanoparticle absorption peak shifts in value depending on the medium permittivity (Figure 7d). The comparison of 30 nm Ga hemisphere with 2 nm oxide shell in THF (*n* = 1.4) and aqueous solution (*n* = 1.33) shows a red-shifted curve in the case of the higher refractive index solution, with absorption peaks displayed at 285 nm and 273 nm, respectively. Later, these results were compared with the measured absorption band and good agreement was observed.

In conclusion, the Ga NPs’ oxide shell likely acts as a hydrophilic material in water, provoking the agglomeration phenomenon. Also, by means of different pH, the ion concentration around the colloidal NPs can be varied and the cluster formation may change because of it.

## 3. Materials and Methods

### 3.1. Ga NPs Synthesis

A colloidal solution of gallium nanoparticles was prepared following the procedure as shown in Figure 1. First, a substrate of silicon with an area of 0.5 cm × 2 cm was cleaved and cleaned using an aqueous solution of 40% hydrofluoric acid in order to remove the native oxide. The AZO [40] expendable layer was deposited using an Alcatel A450 radio frequencies (RF) sputtering system. For the sake of comparison, the layers were prepared depositing at room temperature and 300 °C. The system comprises an RF source set at 150 W and a 50 sccm Ar flow to generate the plasma that sputters the AZO target (Kurt J. Lesker ZnO/Al_2_O_3_, 2% Standard Doping). Prior to deposition, the target was sputtered for 1 min to remove impurities. The deposition time for the AZO layer was 10 min under a pressure of $10^{-2}$ mbar to obtain a 300 nm thick layer. The AZO was chosen taking into account its fast etching in moderately acidic solutions.

Gallium was evaporated [41] on the expendable layer using a Joule-effect Edwards E306 evaporation system (Moorfield, Knutsford, UK). For our process, 0.28 g of solid Ga (99.9999%) was heated for 30 s with a 50 W power under $10^{-6}$ Torr pressure. The distance from the material heater to the sample was set at 20 cm. An ice cooling system was employed to keep the substrate temperature as low as possible, in order to limit the metal surface mobility during the nucleation process. During the growth, the lower the substrate temperature the less NPs coarsening occurs.

To prepare the colloidal solutions, several vials of 3 mL volume were filled with the following solvents: DIW and THF. After Ga deposition and extraction from the thermal evaporation system, the sample was immersed in a phosphoric acid/acetic acid/deionized water (1:1:75) bath for 40 s to partially etch the AZO layer and weaken the structural support of the NPs. Later, it was rinsed in water and promptly inserted into the selected solvent. To allow the NPs transfer from the sample to the solution, an ultrasound treatment of 1′30″ was used.

### 3.2. Scanning Electron Microscopy (SEM)

The SEM investigation was carried out using a Philips XL30 S-FEG microscope (Philips, Amsterdam, The Netherlands) under a $10^{-3}$ bar pressure at room temperature. The colloidal solution was drop cast on a clean silicon substrate, and promptly analyzed in order to avoid possible contamination effects. After the investigation, images were post-processed by Gwyddion open-source software to estimate nanoparticle size and distribution [42].

### 3.3. Atomic Force Microscopy (AFM)

In order to characterize the sputtered AZO layer surface, an Agilent PicoPlus 5500 AFM (Agilent Technologies Inc., Santa Clara, CA, USA) was used operating in dynamic mode. The analyzed samples comprised a 300 nm thick AZO layer grown on a silicon substrate at room temperature and 300 °C. Images were analyzed and post-processed by Gwyddion open source software to investigate the surface morphology of both samples.

### 3.4. pH Measurement

Colloidal sample pH was varied by adding NaOH $10^{-1}$ M and HCl $10^{-1}$ M solutions to achieve the target acidity. The total added liquid did not exceed 0.1% of the original volume (3 mL), in order to avoid changes of the colloid concentration and, therefore, the optical absorption signal. For all solutions, the pH was measured before and after the spectrophotometry analysis to rule out pH changes during measurement due to rearrangement of ions in the solution.

### 3.5. UV-Visible Spectrophotometry

UV-visible spectra of the different colloidal samples were acquired using a Thermo Scientific GO spectrophotometer. For the analysis, differential optical absorption was measured on the colloidal solution poured in a 1.0 cm quartz cuvette using the same colloidal solvent as reference. By means of the equipment, temperature was fixed at 25 °C and exposed to a light ranging from 200 nm to 1000 nm in wavelength, with a system resolution of 1 nm.

## 4. Conclusions

We investigated the properties of the colloidal Ga NPs in THF and aqueous solvents with the use of SEM, AFM and UV spectrophotometer. The colloidal solutions were prepared from the evaporation of nanoparticles on an expandable layer, which gave plenty of possible solutions (such as ion implantation or thermal annealing) for the modification of physico-chemical properties. The AZO expandable layer growth was optimized in order to achieve the highest yield and reproducibility of the synthesis. The NPs’ endurance during the etch step of the process was also proved. The colloidal Ga NPs were surrounded by GaOx, and had different agglomeration status depending on the solvent. In the aqueous solution case, the oxide shell had a hydrophilic behavior and NPs tended to agglomerate promptly, depending on the pH. A broad absorption along the UV-visible spectrum due to cluster formation was observed. In the THF solvent, the gallium oxide shell instead acted as a hydrophobic interface, and colloidal flocculation was mostly prevented. In that case, a strong and quite narrow UV absorption band was measured. Later, colloidal absorption bands were explained by DDA numerical simulations. Jointly with the use of the centrifugation process, it will be possible to select different absorption bands, depending on the evaporated nanoparticles’ dimensions. Future works may use these results to investigate and develop new medical and innovative applications based on Ga NPs.

## Figures and Tables

**Table 1:** Solvent properties. Optical characteristics were extracted from [27].

Solvent	Class	Refractive Index @ 270 nm	Solvent λ Cut-Off (nm)
Water	Polar protic	1.33	190
THF	Polar aprotic	1.40	212
